# Changes in body mass index in Canadians over a five-year period: Results of a prospective, population-based study

**DOI:** 10.1186/1471-2458-7-150

**Published:** 2007-07-09

**Authors:** Wilma M Hopman, Cristine Leroux, Claudie Berger, Lawrence Joseph, Susan I Barr, Jerilynn C Prior, Mark Harrison, Suzette Poliquin, Tanveer Towheed, Tassos Anastassiades, David Goltzman, the CaMos Research Group

**Affiliations:** 1 Clinical Research Centre, Kingston General Hospital and Department of Community Health and Epidemiology, Queen's University, Kingston, Ontario, Canada; 2 CaM*os *Methods Centre, McGill University, Montreal, Quebec, Canada; 3 Department of Epidemiology and Biostatistics, McGill University, Montreal, Quebec, Canada; 4 Human Nutrition, University of British Columbia, Vancouver, British Columbia, Canada; 5 Division of Endocrinology, Department of Medicine, University of British Columbia, British Columbia, Canada; 6 Human Mobility Research Centre and the Division of Orthopedics, Department of Surgery, Queen's University, Kingston, Ontario, Canada; 7 CaM*os *National Coordinating Centre, McGill University, Montreal, Quebec, Canada; 8 Division of Rheumatology, Department of Medicine; Department of Community Health and Epidemiology, Queen's University, Kingston, Ontario, Canada; 9 Division of Rheumatology, Department of Medicine, Queen's University, Kingston, Ontario, Canada; 10 Department of Medicine, McGill University and Calcium Research Laboratory, Royal Victoria Hospital, Montreal, Quebec, Canada

## Abstract

**Background:**

The initiation of the Canadian Multicentre Osteoporosis Study in 1996, and subsequent follow-up of the cohort 5 years later, provided longitudinal body mass index (BMI) data for a random sample of Canadians.

**Methods:**

Height and weight were measured at baseline and 5 years and used to calculate BMI and assign one of six weight categories. Multiple imputation was used to adjust for missing weight at year 5. Data were stratified by age and gender. The proportion of participants moving between categories was generated, and multivariable linear regression was used to identify factors associated with weight change.

**Results:**

Baseline data were available for 8548 participants, year 5 data for 6721, and year 5 weight was imputed for 1827 (17.6%). Mean BMI for every age and gender group exceeded healthy weight guidelines. Most remained within their BMI classification over 5 years, but when change occurred, BMI category was more likely to increase than decrease. Several sociodemographic, lifestyle and clinical characteristics were associated with change.

**Conclusion:**

Mean baseline BMI tended to be higher than recommended. Moreover, on average, men under age 45 and women under age 55 were gaining approximately 0.45 kilograms (one pound) per year, which leveled off with increased age and reversed in the oldest age groups. These findings underscore the need for public health efforts aimed at combating obesity.

## Background

The World Health Organization has recommended an updated classification system for the assessment of body mass index (BMI, defined as weight in kilograms divided by height in meters squared) in adults [1]. This system defines underweight as a BMI of < 18.5, normal as 18.5–24.99, overweight as 25.0–29.99, and includes three classes of obesity for those with a BMI of 30.0–34.99, 35.0–39.99, and ≥ 40.0 [1,2]. Given this new classification, it is helpful to understand changes in BMI over time in the general population.

It is widely accepted that given a certain height, there is a range of weight that is associated with good general health [2,3]. Despite this knowledge, there is evidence that the prevalence of being overweight or obese is reaching epidemic proportions across all age groups, both in developed and developing countries [2,4-11]. The most recent US data based on measured height and weight found that 65.1% of adults over 18 years were overweight [9]. Canadian data based on measured heights and weights found that 65% of men and 53% of women over 18 years were overweight or obese [12], suggesting that the overall US rate is somewhat higher than that of Canadians [9].

Being overweight or obese can have a significant impact on health, as these individuals are more likely to suffer from a variety of illnesses [1,2,10,11,13], have an increased risk of mortality [14-16], and use more health care resources and disability days [11] than their normal weight peers. In 2000, the cost of obesity in the US was estimated at $117 billion dollars [17], while the Canadian estimate for 2001 was $4.3 billion US [18]. Being underweight is also associated with a number of health risks [2,3,19] and an increased risk of mortality [14,16], although considerably less work has gone into assessment of underweight as compared to overweight individuals [2,3].

Few longitudinal studies have assessed changes in BMI over time. Data with measurements at two-year intervals from the Framingham Heart Study suggest that BMI tends to be stable over time [20]. The Canada Fitness Survey, which had an upper age limit of 69 years at baseline, found that weights tended to be reasonably stable over 7 years [21], although all mean seven-year changes were in an upward direction. However, data from the National Population Health Survey (Statistics Canada), with 8 years of follow-up of self-reported BMI, suggest that 32% of those whose weight was normal at baseline became overweight, and that almost a quarter of those who had been overweight became obese, while only 10% of those who were overweight dropped to the normal category [22].

There is a need for a population-based study of measured BMI in order to examine longitudinal weight change [5,7,13]. The initiation of the Canadian Multicentre Osteoporosis Study (CaM*os*) in 1996, and subsequent follow-up of the cohort 5 years later, provided this opportunity. CaM*os *is not a study of individuals with osteoporosis; rather, it was designed to study the incidence and prevalence of osteoporosis in a random sample of Canadians 25 years of age and older [23]. This allowed us to examine the number of participants in each of the weight categories at baseline and 5 years, stratified by age and gender, to determine if BMI is increasing and if so, which age and sex groups are at greatest risk.

## Methods

CaM*os *is an on-going, prospective cohort study of 9,423 non-institutionalized, randomly selected men and women aged 25 years and older at baseline, drawn from a 50-kilometer radius of nine Canadian cities (St John's, Halifax, Quebec City, Toronto, Hamilton, Kingston, Saskatoon, Calgary and Vancouver). Baseline assessments took place between February 1996 and September 1997, and the 5-year follow-up took place between February 2001 and September 2002. All data were obtained by trained interviewers, who used a questionnaire with good reproducibility that was designed for the CaM*os *study. Participants provided written consent.

A detailed description of the objectives, methodology and sampling framework for CaM*os *is available elsewhere [23]. Briefly, households within each region were selected by random draws of listed telephone numbers, and one randomly selected household member 25 years of age or older was asked to participate. Of 22,173 eligible households, 27.5% declined to participate, 30.0% completed a short questionnaire that provided information about the age, gender and fracture history of the residents, and 9,423 (42.5%) went on to participate fully in the study. CaM*os *was designed to collect epidemiological data related to the incidence and prevalence of osteoporosis, so although the sampling framework was random, it was designed to include more women than men, and a higher number of older than younger Canadian residents.

Ethics approval was obtained through the Review Boards of each participating centre. These included Memorial University of Newfoundland Human Investigation Committee (St John's), the Capital Health Research Ethics Board (Halifax), Comité d'éthique de la recherché clinique du Centre Hopitalier Universitaire de Québec (Quebec City), St. Michael's Hospital Research Ethics Board (Toronto), McMaster University Research Ethics Board (Hamilton), Queen's University Health Sciences and Affiliated Teaching Hospitals Research Ethics Board (Kingston), University of Saskatchewan Biomedical Research Ethics Board (Saskatoon), University of Calgary Health Research Ethics Board (Calgary), University of British Columbia Clinical Research Ethics Board (Vancouver) and the McGill University Health Centre Research Ethics Committee (Montreal).

The majority of participants were scheduled for dual-energy x-ray absorptiometry (DXA) assessment of bone mineral density (BMD) as part of the study protocol, at which time both height and weight were measured. Height was measured without shoes, using a wall-mounted stadiometer, ruler on the wall or a measure on a weight scale. Weight was measured in light clothing using a beam balance or electronic scale. For those who elected not to have the DXA, or for whom it could not be scheduled, the interviewer measured height and weight with a carpenter's rule and a portable scale. Baseline height and weight were used to calculate the baseline BMI. Five-year BMI was calculated using the baseline height and the weight at year 5. Baseline height was used to avoid the need to impute height for those missing height at year 5, as the loss of height over time was expected to be close to zero for the majority of participants. Change in BMI was calculated as year 5 minus the baseline value, so that positive values would indicate an increase in weight.

Multivariable linear regression was used to identify the factors associated with five year changes in BMI for men and women. Variables were identified on the basis of an extensive literature review, and included sociodemographic characteristics (age, region of Canada, education, annual household income, whether they lived alone and employment status), clinical characteristics (menopausal status, parity, a variety of individual comorbid conditions, and number of comorbid conditions), and other factors (smoking, alcohol use, hours spent walking, hours spent sitting, participation in regular activities, level of pain, level of happiness and perception of general health). In addition, since we expected that changes in some of these variables could be associated with changes in BMI, change variables were also examined for menopausal status, comorbidities, smoking, alcohol use, the activity variables, level of pain, level of happiness, and perception of general health.

Univariate associations between all variables and change in BMI were examined to refine the list of predictors identified above, removing any for which there was no evidence to support its inclusion. For some, the 95% confidence interval (CI) may have included zero but if one or both of the upper or lower limits could be of clinical interest, the variable was retained, allowing the investigators to see the effects of all variables of potential interest. Three variables not automatically retained in the model by these *a priori *considerations (menopausal status, parity and alcohol consumption) were retained due to widespread belief that the variable was associated with weight change [22,24-26].

Not all participants who provided baseline data participated at year 5, and differences in the characteristics between participants and non-participants could cause the estimates to be biased. For example, this could occur if subjects with certain diseases tended to lose weight or not participate because they were unwell. Multiple imputation [27] was therefore used to at least partially adjust for possible selection bias for all surviving participants who did not provide 5-year data. In addition, those who were pregnant at baseline or year 5 were excluded from the analysis, as were those who did not have height and/or weight recorded at baseline.

Within the multiple imputation models, linear regression models were fit to predict weight at year 5, using data from respondents with complete data at both time points. Models included baseline height and weight, age, and BMD of the femoral neck from year 5 if available, otherwise at baseline. Separate models were developed for men and women within each age group. Covariate data from those missing year 5 weights were then entered into the models to impute the missing weights. Simultaneous regression parameter estimations and multiple imputations were carried out via a Gibbs sampler algorithm as implemented in WinBugs software (version 1.4, Cambridge, Institute of Public Health, MRC Biostatistics Unit, 2004).

## Results

Complete baseline data were available for 2572 men and 5976 women, for a total of 8548 (90.7% of the original cohort). Height and/or weight were missing for 277 at baseline, primarily due to scheduling difficulties for the DXA at two sites. Six were pregnant at baseline or at follow-up. Six hundred and forty-four had died by the time of the 5-year follow-up, and although 592 of these had provided baseline data (52 of those who died were also missing the baseline measurements), their data were not used since 5-year BMI changes were undefined for these subjects.

At year 5, data were available for 1959 (76.2%) men and 4762 (79.7%) women, for a total of 6721, and were imputed for 1827 (613 men and 1214 women). Of these 1827, 764 were still in the cohort but were missing weight at year 5, largely due to scheduling difficulties and one site that had not measured weight at the time of the DXA. Another 415 completed a short questionnaire over the telephone. The reasons for loss to follow-up included 265 who could not be contacted, 106 who withdrew consent, 89 who were too sick, 114 who had moved away, and 74 with miscellaneous reasons such as cancelled, no time, or no reason given.

Those without complete year 5 were represented in all categories of weight. For men, 33.3% of those in the underweight group and 35.0% of those in the obese class III group at baseline were missing year 5 data. For other weight categories, the proportions with incomplete year 5 data ranged from 21.4% (overweight) to 26.3% (obese class I). Among women, the proportions with incomplete data ranged from 19.1% of those in the normal weight category to 27.8% of those in obese class III. For the other categories, the proportions were very similar, ranging from 20.5% (overweight) to 21.8 (obese class II), suggesting that there was no bias due to greater loss to follow-up within certain weight categories.

**Figure 1:**
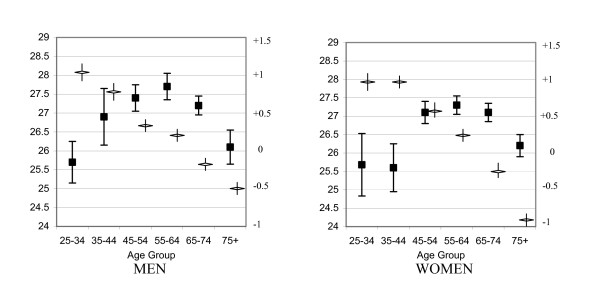
**Baseline body mass index (BMI) and 5-year change in BMI, by age group and gender**. Numbers on the left axis represent body mass index (BMI). Solid squares represent the mean baseline BMI, and the error bars represent the 95% confidence interval (CI). Numbers on the right axis represent mean change in BMI. Open triangles represent the mean 5-year change in BMI, and the error bars represent the 95% CI.

Figure 1 contains the mean baseline BMI for men and women across 10-year age stratifications, along with the associated 95% confidence intervals (CI). The mean BMI for every age group for both genders exceeded the healthy weight guidelines of BMI below 25^1,2^. The lower limit of the 95% CI was less than 25 for only one group (women 25–34 years), and was exactly 25.0 for women aged 35–44 years. This figure also contains the mean 5-year change in BMI with the 95% CI for the age and gender groups. The increases in BMI were much larger for men between 25 and 44 years and women between 25 and 54 years than in the older groups, in which the BMI tended to decrease.

Tables 1 (men) and 2 (women) contain the weight classifications at baseline by age group, along with the Year 5 values (actual and imputed). For men, with the exception of the baseline weight for the 25–34 year age group and the Year 5 observed and imputation adjusted values for the 75+ age group, the majority of values fell into the overweight category. For women, the majority of values were in the normal category for the two youngest groups and the 75+ age group. For those aged 45–74 years, the percentages in the normal weight and overweight categories were very close. A small majority of those in the 45–54 year age group were in the normal weight category, while an equally small majority of those aged 55–74 years were in the overweight category.

**Table 1:** Weight classification at baseline and year five (including imputed) for men. Values are expressed as frequencies and percent of age group total

Baseline Age	Time	Sample Size*	Underweight	Normal Weight	Overweight	Obese Class I	Obese Class II	Obese Class III
25 – 34	Baseline Year 5	187 187	2 (1.1) 1 (0.5)	85 (45.5) 70 (37.4)	78 (41.7) 82 (43.9)	18 (9.3) 25 (13.4)	3 (1.6) 7 (3.7)	1 (0.5) 2 (1.1)
35 – 44	Baseline Year 5	205 205	2 (1.0) 1 (0.5)	70 (34.2) 53 (25.9)	97 (47.3) 106 (51.7)	28 (13.7) 34 (16.6)	5 (2.4) 6 (2.9)	3 (1.5) 5 (2.4)
45 – 54	Baseline Year 5	564 565	1 (0.2) 3 (0.5)	157 (27.8) 145 (25.7)	288 (51.1) 277 (49.0)	89 (15.8) 104 (18.4)	25 (4.4) 31 (5.5)	4 (0.7) 5 (0.9)
55 – 64	Baseline Year 5	609 608	2 (0.3) 2 (0.3)	135 (22.2) 132 (21.7)	317 (52.1) 308 (50.7)	125 (20.5) 129 (21.2)	26 (4.3) 33 (5.4)	4 (0.7) 4(0.7)
65 – 74	Baseline Year 5	705 705	3 (0.4) 3 (0.4)	197 (27.9) 220 (31.2)	361 (51.2) 344 (48.8)	127 (18.0) 117 (16.6)	11 (1.6) 17 (2.4)	6 (0.9) 4 (0.6)
75 +	Baseline Year 5	302 302	2 (0.7) 6 (2.0)	117 (38.7) 137 (45.4)	143 (47.4) 116 (38.4)	32 (10.6) 36 (11.9)	6 (2.0) 5 (1.7)	2 (0.7) 2 (0.7)

* Baseline and year 5 values are not always identical due to rounding in the imputation process.

Underweight = BMI<18; Normal weight = BMI 18.5–24.99; Overweight = BMI 25.0–29.99; Obese Class I = BMI 30.0–34.99; Obese Class II = BMI 35.0–39.99; Obese Class III = BMI ≥ 40.0

**Table 2:** Weight classification at baseline and year five (including imputed) for women. Values are expressed as frequencies and percent of age group total

Baseline Age	Time	Sample Size*	Underweight	Normal Weight	Overweight	Obese Class I	Obese Class II	Obese Class III
25 – 34	Baseline Year 5	187 188	5 (2.7) 4 (2.1)	101 (54.0) 84 (44.7)	50 (26.7) 56 (29.8)	15 (8.0) 25 (13.3)	10 (5.4) 11 (5.9)	6 (3.2) 8 (4.3)
35 – 44	Baseline Year 5	278 277	3 (1.1) 3 (1.1)	155 (55.8) 140 (50.5)	70 (25.2) 66 (23.8)	34 (12.2) 44 (15.9)	7 (2.5) 16 (5.8)	9 (3.2) 8 (2.9)
45 – 54	Baseline Year 5	1082 1083	10 (0.9) 12 (1.1)	433 (40.0) 382 (35.3)	377 (34.8) 382 (35.3)	158 (14.6) 189 (17.5)	81 (7.5) 79 (7.3)	23 (2.1) 39 (3.6)
55 – 64	Baseline Year 5	1564 1562	19 (1.2) 28 (1.8)	526 (33.6) 469 (30.0)	629 (40.2) 623 (39.9)	269 (17.2) 315 (20.2)	87 (5.6) 92 (5.9)	34 (2.2) 35 (2.2)
65 – 74	Baseline Year 5	1945 1940	37 (1.9) 56 (2.9)	680 (35.0) 692 (35.7)	735 (37.8) 713 (36.8)	362 (18.6) 352 (18.1)	99 (5.1) 99 (5.1)	32 (1.7) 28 (1.4)
75 +	Baseline Year 5	920 918	24 (2.6) 49 (5.3)	367 (39.9) 414 (45.1)	351 (38.2) 317 (34.5)	142 (15.4) 113 (12.3)	32 (3.5) 22 (2.4)	4 (0.4) 3 (0.3)

* Baseline and year 5 values are not always identical due to rounding in the imputation process.

Underweight = BMI<18; Normal weight = BMI 18.5–24.99; Overweight = BMI 25.0–29.99; Obese Class I = BMI 30.0–34.99; Obese Class II = BMI 35.0–39.99; Obese Class III = BMI ≥ 40.0

Tables 3 (men) and 4 (women) contain the percentages staying within a certain BMI category or moving into another group, by age. For example, a man in his early 40's and of normal weight at baseline has a 62.7% chance of staying within his weight category, a 0.5% chance of dropping into the underweight category and a 34.4% chance of moving into the overweight category after 5 years. The largest probabilities are within the same weight category at baseline and at year 5, suggesting that people are most likely to remain within their BMI classifications. The major exceptions to this are in underweight men in most age groups (Table 3), where weight gain into the normal range generally occurred. Furthermore, in general, the data suggest that if there is a change, there is a far greater probability of increasing by a category compared to decreasing by a category in the younger age groups; this reverses for the older age groups and at higher levels of weight.

**Table 3:** Percentage of men staying within baseline weight category versus switching to another category

Age at baseline(years)	Category at Baseline	Underweight At Year 5 (95% CI)	Normal Weight At Year 5 (95% CI)	Overweight At Year 5 (95% CI)	Obese Class I At Year 5 (95% CI)	Obese Class II At year 5 (95% CI)	Obese Class III At Year 5 (95% CI)
25 – 34	Underweight	21.2 (0.0–77.3)	60.5 (8.6–100.0)	3.9 (0.0–25.0)	3.6 (0.0–22.1)	3.4 (0.0–21.8)	7.4 (0.0–39.3)
	Normal Weight	1.2 (0.0–4.5)	67.9 (57.0–78.7)	30.3 (19.9–41.0)	0.2 (0.0–1.3)	0.1 (0.0–0.6)	0.2 (0.0–1.2)
	Overweight	0.1 (0.0–0.7)	13.1 (5.8–21.4)	71.1 (59.4–82.2)	15.2 (6.3–24.5)	0.2 (0.0–1.4)	0.2 (0.0–1.3)
	Obese Class I	0.5 (0.0–3.1)	0.5 (0.0–3.3)	3.5 (0.0–15.6)	68.2 (44.0–90.7)	26.0 (5.2–48.3)	1.2 (0.0–6.2)
	Obese Class II	2.6 (0.0–16.2)	2.7 (0.0–16.3)	2.7 (0.0–17.1)	5.9 (0.0–36.6)	50.2 (5.8–94.9)	35.9 (0.1–80.0)
	Obese Class III	5.8 (0.0–37.5)	5.7 (0.0–36.6)	6.0 (0.0–39.8)	5.8 (0.0–38.4)	6.1 (0.0–38.5)	70.6 (14.4–100.0)

35 – 44	Underweight	18.1 (0.0–74.9)	62.9 (9.5–100.0)	4.2 (0.0–27.2)	3.7 (0.0–24.1)	3.7 (0.0–23.1)	7.3 (0.0–37.1)
	Normal Weight	0.5 (0.0–2.8)	62.7 (49.6–76.5)	34.4 (21.3–47.9)	0.6 (0.0–3.0)	1.6 (0.0–4.5)	0.3 (0.0–1.5)
	Overweight	0.1 (0.0–0.6)	7.3 (2.0–13.5)	79.1 (69.6–87.9)	13.0 (6.0–20.9)	0.2 (0.0–1.0)	0.2 (0.0–1.1)
	Obese Class I	0.3 (0.0–2.1)	0.4 (0.0–2.4)	17.2 (3.5–32.9)	73.1 (54.2–90.7)	8.3 (0.0–20.0)	0.7 (0.0–3.7)
	Obese Class II	1.8 (0.0–11.0)	1.8 (0.0–10.8)	1.8 (0.0–10.7)	2.3 (0.0–14.3)	49.3 (11.3–87.8)	43.0 (6.3–81.1)
	Obese Class III	2.6 (0.0–16.5)	2.7 (0.0–16.7)	2.9 (0.0–17.9)	2.7 (0.0–16.9)	8.1 (0.0–46.4)	81.0 (37.0–100.0)

45 – 54	Underweight	36.3 (0.0–98.2)	34.3 (0.0–98.1)	5.9 (0.0–38.7)	5.7 (0.0–37.6)	6.0 (0.0–38.3)	11.8 (0.0–58.1)
	Normal Weight	1.4 (0.0–3.5)	73.9 (66.1–81.4)	23.9 (16.7–31.5)	0.7 (0.0–2.1)	0.1 (0.0–0.4)	0.1 (0.0–0.6)
	Overweight	0.0 (0.0–0.2)	9.9 (6.1–13.7)	76.6 (71.4–82.0)	13.0 (8.8–17.2)	0.4 (0.0–1.1)	0.1 (0.0–0.4)
	Obese Class I	0.1 (0.0–0.7)	0.1 (0.0–0.7)	19.8 (11.5–29.4)	68.7 (57.9–79.1)	11.1 (4.4–18.3)	0.2 (0.0–1.1)
	Obese Class II	0.4 (0.0–2.3)	0.4 (0.0–2.3)	4.3 (0.0–12.1)	12.4 (0.7–26.1)	72.1 (53.9–90.3)	10.4 (0.9–23.0)
	Obese Class III	2.1 (0.0–13.4)	2.1 (0.0–13.2)	2.1 (0.0–13.3)	2.2 (0.0–13.6)	34.5 (0.0–77.3)	56.9 (15.4–99.0)

55 – 64	Underweight	40.7 (0.3–85.2)	40.9 (0.1–85.6)	3.8 (0.0–23.8)	3.7 (0.0–23.2)	3.6 (0.0–23.2)	7.4 (0.0–39.3)
	Normal Weight	0.2 (0.0–1.4)	74.0 (66.2–81.6)	24.7 (17.4–32.6)	0.9 (0.0–2.5)	0.1 (0.0–0.5)	0.2 (0.0–0.8)
	Overweight	0.0 (0.0–0.2)	9.7 (6.3–13.5)	78.0 (72.9–82.9)	12.2 (8.4–16.1)	0.0 (0.0–0.3)	0.1 (0.0–0.3)
	Obese Class I	0.9 (0.0–2.5)	0.1 (0.0–0.6)	21.5 (13.6–29.5)	67.1 (58.0–76.1)	10.3 (5.0–16.4)	0.2 (0.0–0.9)
	Obese Class II	0.4 (0.0–2.3)	0.4 (0.0–2.2)	0.5 (0.0–2.6)	16.0 (2.8–30.5)	72.8 (54.4–89.5)	10.0 (0.6–21.7)
	Obese Class III	2.2 (0.0–13.4)	2.1 (0.0–12.8)	2.1 (0.0–12.7)	23.8 (0.0–59.2)	29.3 (0.0–76.9)	40.5 (0.0–85.5)

65 – 74	Underweight	29.6 (0.0–69.7)	57.1 (16.4–96.5)	2.5 (0.0–15.7)	2.8 (0.0–17.1)	2.6 (0.0–16.4)	5.4 (0.0–27.6)
	Normal Weight	0.5 (0.0–1.8)	86.2 (80.7–91.5)	13.1 (8.0–18.5)	0.1 (0.0–0.4)	0.1 (0.0–0.3)	0.1 (0.0–0.5)
	Overweight	0.3 (0.0–0.9)	13.1 (9.3–16.8)	80.1 (75.4–84.4)	6.5 (3.7–9.2)	0.0 (0.0–0.2)	0.1 (0.0–0.3)
	Obese Class I	0.1 (0.0–0.5)	0.9 (0.0–2.6)	21.3 (13.8–29.4)	71.3 (62.5–79.7)	6.2 (2.2–11.0)	0.2 (0.0–0.8)
	Obese Class II	0.8 (0.0–5.0)	0.9 (0.0–5.0)	9.5 (0.0–26.1)	24.2 (2.3–49.6)	62.3 (34.8–90.6)	2.3 (0.0–12.2)
	Obese Class III	1.5 (0.0–8.6)	1.5 (0.0–9.2)	1.5 (0.0–9.4)	1.5 (0.0–8.7)	31.4 (3.0–63.4)	62.7 (29.2–93.9)

75 +	Underweight	62.6 (9.0–100.0)	18.8 (0.0–74.9)	3.8 (0.0–23.6)	3.6 (0.0–22.9)	3.8 (0.0–23.4)	7.4 (0.0–38.4)
	Normal Weight	4.0 (0.2–8.5)	87.3 (80.0–94.3)	7.5 (2.2–13.4)	0.9 (0.0–2.6)	0.1 (0.0–0.5)	0.2 (0.0–0.9)
	Overweight	0.1 (0.0–0.4)	24.3 (16.5–32.3)	68.7 (60.0–77.0)	6.7 (2.4–11.3)	0.1 (0.0–0.4)	0.1 (0.0–0.8)
	Obese Class I	0.3 (0.0–1.7)	0.4 (0.0–2.2)	23.2 (7.6–38.6)	70.1 (52.6–86.6)	5.4 (0.0–14.4)	0.6 (0.0–3.2)
	Obese Class II	1.5 (0.0–9.1)	1.5 (0.0–8.8)	16.5 (0.0–43.2)	35.5 (0.1–74.4)	40.5 (0.0–78.0)	4.5 (0.0–23.1)
	Obese Class III	3.7 (0.0–23.7)	3.5 (0.0–22.5)	3.8 (0.0–24.3)	3.7 (0.0–23.7)	18.2 (0.0–74.8)	67.0 (12.2–100.0)

CI = Confidence Interval

**Table 4:** Percentage of women staying within baseline weight category versus switching to another category

Age at baseline(years)	Category at Baseline	Underweight At Year 5 (95% CI)	Normal Weight At Year 5 (95% CI)	Overweight At Year 5 (95% CI)	Obese Class I At Year 5 (95% CI)	Obese Class II At year 5 (95% CI)	Obese Class III At Year 5 (95% CI)
25 – 34	Underweight	54.4 (18.1–89.8)	36.8 (3.4–72.2)	1.7 (0.0–10.4)	1.8 (0.0–11.3)	1.8 (0.0–10.7)	3.5 (0.0–18.2)
	Normal Weight	1.0 (0.0–3.7)	75.5 (65.9–84.4)	22.8 (13.7–31.8)	0.4 (0.0–2.0)	0.1 (0.0–0.5)	0.2 (0.0–1.0)
	Overweight	0.2 (0.0–1.1)	10.5 (2.6–19.7)	56.9 (41.5–71.4)	29.5 (16.2–43.2)	2.5 (0.0–7.0)	0.4 (0.0–2.0)
	Obese Class I	0.6 (0.0–3.5)	0.7 (0.0–4.2)	24.5 (4.5–47.1)	51.6 (24.9–75.8)	8.6 (0.0–23.7)	14.0 (0.7–30.7)
	Obese Class II	0.9 (0.0–5.5)	1.0 (0.0–5.9)	1.0 (0.0–5.6)	14.6 (0.0–37.7)	57.7 (29.0–88.6)	24.8 (1.8–50.2)
	Obese Class III	1.5 (0.0–9.2)	1.5 (0.0–9.1)	1.5 (0.0–9.0)	1.6 (0.0–9.3)	34.5 (3.1–68.5)	59.3 (23.8–92.3)

35 – 44	Underweight	2.5 (0.0–15.5)	84.1 (48.6–100.0)	2.6 (0.0–15.4)	2.7 (0.0–16.5)	2.7 (0.0–16.9)	5.4 (0.0–28.3)
	Normal Weight	2.1 (0.1–4.6)	82.7 (76.3–89.1)	13.6 (7.8–19.3)	1.4 (0.1–3.4)	0.1 (0.0–0.4)	0.1 (0.0–0.7)
	Overweight	0.2 (0.0–0.9)	12.5 (4.6–21.4)	58.3 (45.2–70.6)	28.5 (17.8–40.4)	0.3 (0.0–1.9)	0.3 (0.0–1.6)
	Obese Class I	0.3 (0.0–1.6)	0.3 (0.0–2.1)	12.5 (2.1–24.9)	59.8 (42.0–77.6)	26.2 (10.9–42.8)	0.9 (0.0–4.5)
	Obese Class II	1.3 (0.0–7.6)	1.4 (0.0–8.3)	1.4 (0.0–8.0)	20.9 (0.0–52.3)	58.9 (22.4–96.4)	16.3 (0.0–47.8)
	Obese Class III	1.0 (0.0–5.8)	1.0 (0.0–5.9)	1.1 (0.0–6.5)	1.0 (0.0–6.3)	24.3 (0.9–50.3)	71.6 (43.4–96.5)

45 – 54	Underweight	56.9 (28.8–84.3)	38.5 (12.3–65.9)	0.9 (0.0–5.6)	1.0 (0.0–6.1)	0.9 (0.0–5.1)	1.9 (0.0–9.9)
	Normal Weight	1.4 (0.3–2.7)	78.7 (74.5–82.8)	19.3 (15.3–23.1)	0.6 (0.0–1.3)	0.0 (0.0–0.1)	0.0 (0.0–0.2)
	Overweight	0.0 (0.0–0.2)	9.7 (6.6–13.2)	68.2 (62.8–73.2)	20.7 (16.4–25.3)	1.4 (0.4–2.7)	0.1 (0.0–0.3)
	Obese Class I	0.1 (0.0–0.4)	0.7 (0.0–2.1)	21.3 (14.5–28.4)	61.3 (53.2–69.5)	15.8 (9.7–22.0)	0.8 (0.0–2.2)
	Obese Class II	0.1 (0.0–0.7)	0.1 (0.0–0.7)	7.5 (2.6–13.4)	14.4 (6.4–22.8)	54.7 (43.0–65.7)	23.1 (14.0–32.6)
	Obese Class III	0.4 (0.0–2.6)	0.4 (0.0–2.4)	0.4 (0.0–2.6)	0.6 (0.0–3.7)	17.8 (1.8–35.4)	80.4 (62.3–96.8)

55 – 64	Underweight	51.2 (28.4–74.2)	46.3 (23.5–68.9)	0.5 (0.0–3.0)	0.5 (0.0–2.9)	0.5 (0.0–3.1)	1.0 (0.0–5.3)
	Normal Weight	3.0 (1.5–4.7)	73.9 (69.8–77.7)	22.4 (18.7–26.2)	0.6 (0.1–1.4)	0.0 (0.0–0.1)	0.0 (0.0–0.2)
	Overweight	0.3 (0.0–0.8)	11.1 (8.4–13.8)	71.5 (67.6–75.3)	16.9 (13.9–20.2)	0.2 (0.0–0.6)	0.0 (0.0–0.2)
	Obese Class I	0.0 (0.0–0.2)	0.8 (0.0–1.9)	19.5 (14.5–24.7)	68.1 (62.0–74.1)	10.7 (6.8–14.9)	0.8 (0.0–1.9)
	Obese Class II	0.1 (0.0–0.7)	1.2 (0.0–3.7)	2.5 (0.1–5.8)	23.4 (14.3–33.5)	59.1 (47.8–70.0)	13.6 (6.3–21.2)
	Obese Class III	0.3 (0.0–1.6)	0.3 (0.0–1.7)	0.3 (0.0–1.7)	6.1 (0.4–14.1)	31.0 (15.7–46.6)	62.0 (45.3–77.5)

65 – 74	Underweight	73.2 (58.8–87.6)	25.5 (12.2–40.7)	0.3 (0.0–1.6)	0.3 (0.0–1.6)	0.3 (0.0–1.6)	0.5 (0.0–2.7)
	Normal Weight	3.8 (2.2–5.4)	82.1 (78.9–85.2)	13.3 (10.6–16.1)	0.6 (0.1–1.3)	0.2 (0.0–0.5)	0.0 (0.0–0.2)
	Overweight	0.4 (0.0–0.9)	16.7 (13.8–19.5)	73.1 (69.5–76.6)	9.5 (7.1–11.8)	0.3 (0.0–0.7)	0.0 (0.0–0.1)
	Obese Class I	0.0 (0.0–0.2)	1.2 (0.2–2.3)	21.8 (17.3–26.6)	69.6 (64.5–74.7)	6.7 (4.1–9.6)	0.6 (0.0–1.4)
	Obese Class II	0.1 (0.0–0.6)	0.1 (0.0–0.5)	7.2 (2.5–12.1)	26.4 (16.8–35.5)	61.9 (51.0–71.6)	4.3 (0.7–8.8)
	Obese Class III	0.3 (0.0–1.8)	0.3 (0.0–2.0)	0.3 (0.0–1.9)	0.5 (0.0–2.9)	30.6 (14.3–47.4)	68.0 (50.5–83.9)

75 +	Underweight	76.4 (55.5–94.3)	21.6 (2.9–40.8)	0.4 (0.0–2.6)	0.4 (0.0–2.2)	0.4 (0.0–2.6)	0.8 (0.0–4.2)
	Normal Weight	8.2 (5.1–11.7)	82.0 (77.4–86.5)	9.6 (6.4–13.1)	0.1 (0.0–0.5)	0.0 (0.0–0.2)	0.1 (0.0–0.3)
	Overweight	0.1 (0.0–0.4)	29.9 (24.3–35.2)	63.1 (57.2–68.8)	6.8 (4.1–9.7)	0.0 (0.0–0.2)	0.1 (0.0–0.3)
	Obese Class I	0.1 (0.0–0.4)	2.1 (0.1–4.8)	41.1 (31.7–49.8)	53.8 (44.6–63.0)	2.7 (0.4–5.7)	0.1 (0.0–0.7)
	Obese Class II	0.3 (0.0–1.9)	0.3 (0.0–1.7)	7.1 (0.4–16.4)	39.7 (21.9–59.1)	50.8 (32.2–70.1)	1.8 (0.0–8.0)
	Obese Class III	2.1 (0.0–12.2)	2.2 (0.0–13.2)	2.1 (0.0–12.7)	3.4 (0.0–21.3)	27.0 (0.0–70.9)	63.3 (20.2–100.0)

CI = Confidence Interval

The results of movement from one BMI category to another over 5 years are very similar for women (Table 4). A woman in her early 40's with a normal baseline weight has an 82.7% chance of staying in that category, a 2.1% chance of dropping into the underweight group and a 13.6% chance of moving into the overweight category. However, with increasing age and/or for those in higher BMI categories, there is an increased likelihood of dropping by a category relative to increasing by a category.

Table 5 contains the results of the regression modeling. Thirteen variables were retained in the model for women, while 11 were retained for men. Eight of these (7 for men) were measured at baseline and 5 (4 for men) were based on change between baseline and follow-up. The models accounted for just under 9% of the total variation in outcome, so much of the variance is unexplained by our covariates. In general, variables associated with an increase in weight included household income greater than $20,000, and education levels lower than a university degree. All regions of Canada were associated with less gain, as compared to the reference category (Saskatoon, representing Central Canada). All age categories for both genders were associated with weight gain when compared to the reference category of age 75+, as already demonstrated in Figure 1.

**Table 5:** Multivariable linear regression models for change in BMI for women and men

Variable	Women Parameter Estimates and 95% Confidence Intervals	Men Parameter Estimates and 95% Confidence Intervals
Intercept	3.6 (2.8, 4.3)	1.2 (0.5, 2.0)

Region of Canada (Reference = Central)		
East	-0.82 (-1.09, -0.55)	-0.66 (-0.99, -0.32)
Quebec	-0.50 (-0.78, -0.22)	-0.54 (-0.88, -0.20)
Ontario	-0.39 (-0.61, -0.16)	-0.35 (-0.62, -0.08)
West	-0.24 (-0.47, -0.00)	-0.45 (-0.73, -0.16)

Age at baseline (Reference = 75+)		
25–34	1.97 (0.23, 3.71)	1.55 (1.10, 2.00)
35–44	2.13 (1.59, 2.68)	1.19 (0.76, 1.63)
45–54	1.64 (1.34, 1.94)	0.75 (0.40, 1.10)
55–64	1.11 (0.88, 1.35)	0.76 (0.43, 1.09)
65–74	0.69 (0.46, 0.92)	0.41 (0.09, 0.73)

Annual Household Income (Reference = < $20,000)		
$21,000–$40,000	0.13 (-0.07, 0.33)	0.28 (-0.04, 0.60)
$41,000–$60,000	0.08 (-0.16, 0.32)	0.44 (0.10, 0.78)
$61,000–$80,000	0.44 (0.13, 0.74)	0.36 (-0.01, 0.74)
> $80,000	0.18 (-0.12, 0.48)	0.65 (0.27, 1.02)

Education (Reference = University degree(s))		
< High school diploma	0.31 (0.06, 0.55)	0.12 (-0.14, 0.39)
High school or Trade school diploma	0.25 (0.02, 0.48)	0.32 (0.07, 0.56)
Some University, or University certificate/diploma	0.36 (0.08, 0.64)	0.10 (-0.20, 0.40)

Average level of pain, past week (reference = no pain)		
Mild to moderate, prevents no activities	0.19 (0.01, 0.37)	0.11 (-0.11, 0.33)
Moderate, prevents a few activities	0.08 (-0.12, 0.28)	-0.12 (-0.40, 0.16)
Moderate to severe, prevents some activities	0.34 (0.06, 0.63)	0.02 (-0.39, 0.43)
Severe pain that prevents most activities	0.00 (-0.66, 0.67)	0.04 (-0.76, 0.85)

Smoking status (reference = quit smoking)		
Current smoker	-0.49 (-0.74, -0.24)	-0.18 (-0.46, 0.09)
Never smoked	-0.12 (-0.27; 0.04)	-0.01 (-0.21, 0.19)

Number of live births	-0.02 (-0.06, 0.02)	n/a

Alcohol/week (Reference = None)		
1 to 6 (WOMEN) or 1 to 13 (MEN)	0.06 (-0.10, 0.21)	-0.09 (-0.30, 0.13)
7 or more (WOMEN) or 14 or more (MEN)	-0.16 (-0.39, 0.07)	-0.25 (-0.55, 0.05)

Menopausal status (reference = menopaused at baseline)		
Not menopaused	-0.16 (-0.50, 0.17)	n/a
Menopaused in the past 5 years	-0.15 (-0.49, 0.20)	

Change, participation in regular activity (reference = did not, do now)		
Did not, and do not now (no, no)	0.28 (0.05, 0.52)	0.42 (0.12, 0.71)
Did, but do not anymore (yes, no)	0.28 (0.04, 0.53)	0.27 (-0.04, 0.58)
Did and still do (yes, yes)	0.24 (0.01, 0.47)	0.17 (-0.11, 0.45)

Change, number of sedentary hours (reference = high to low)		
Low to high	0.32 (0.09, 0.56)	0.52 (0.22, 0.83)
High at baseline, stayed high	0.13 (-0.08, 0.33)	0.23 (-0.03, 0.49)
Low at baseline, stayed low	0.17 (-0.04, 0.38)	0.38 (0.13, 0.64)

Change in how happy they usually feel (reference = improved)		
Decline in how happy they usually feel	-0.37 (-0.65, -0.10)	-0.33 (-0.66, 0.01)
Feel about the same	-0.23 (-0.44, -0.02)	-0.09 (-0.35, 0.17)

Perception of general health (reference = poor, and stayed that way)		
High, dropped to poor	0.38 (0.15, 0.60)	0.13 (-0.17, 0.43)
Poor, went to high	0.02 (-0.22, 0.27)	0.29 (0.04, 0.53)
Perception of health stayed high	0.12 (-0.06, 0.31)	0.11 (-0.12, 0.33)

The first eight variables are based on baseline data; the remaining 5 represent change between baseline and the 5-year follow-up.

Current smokers and those who had never smoked tended to gain less weight as compared to those who had quit smoking, although only one of the four comparisons attained clinical importance in that the 95% CI did not include zero. Higher alcohol consumption was associated with small declines for both men and women, but the results were inconclusive for both. Those who felt less happy or whose happiness assessment stayed about the same, also saw less gain as compared to those who indicated that their level of happiness had improved, although this result was inconclusive for men. Some categories of changes in participation in regular activity, change in number of sedentary hours, and perception of overall health were also associated with increases in BMI for either men or women, although not always in the expected direction. For example, those who indicated that their number of sedentary hours was quite low still saw a small increase in the parameter estimates.

For women, the association between number of live births and weight change was virtually non-existent. Finally, after adjusting for age, the effect of menopause was also inconclusive, although it is of clinical interest that those who had not yet become menopausal, or who had become menopausal recently, saw small declines as compared to those who were already menopausal at baseline.

## Discussion

These results add to the growing body of literature suggesting that the prevalence of overweight and obesity is increasing. The mean baseline BMI exceeded 25.0 for every age and gender category studied, ranging from 25.6 (women aged 35–44) to 27.7 (men aged 55–64). The mean change over 5 years does not appear large at face value, ranging from an increase of 1.07 for the youngest group of men to a drop of 0.96 for the oldest group of women. However, if one adds the mean change to the mean baseline value, all values remain greater than 25, and the values increase for all but the two oldest age groups, for both men and women. In view of the fact that the mean BMI exceeded 25 even in the youngest age groups in both men and women (Figure 1), it seems likely that excess weight gain has its inception before age 25.

Weight regulation depends on a balance between energy intake via food and energy expenditure. Evolving knowledge indicates that complex neuronal and hormonal input to the hypothalamus and brain stem controls food intake, and peripheral circulating adipostat factors and gut hormones are highly important in appetite control and food intake regulation [28]. The results presented in Tables 1 and 2 suggest that the number within the BMI categories remains relatively stable despite the trend towards small annual increases as seen in Figure 1, which is consistent with previous research [20,21] and the well-developed homeostatic role for weight regulation [28]. The results in Tables 3 and 4 suggest that the highest percentage of those studied will remain within their original weight classification after 5 years. Only in men who were initially underweight was there a tendency to increase BMI into the normal range. This observation may be consistent with the thesis that the complex neurohormonal regulation of weight regulation may have evolved primarily to prevent starvation [28].

In considering the relative stability of the weight categories despite evidence of increasing weight over time, one must bear in mind that a person can stay within their weight category despite a significant weight gain. For example, a woman at the average Canadian height of 163 centimetres (cm) would weigh 49.1 kg for a BMI of 18.50 and 66.3 kg for a BMI of 25.0, representing an increase of 17.2 kg (37.9 pounds) to move into the overweight category if starting at the lowest point in the normal weight category. To do the same, a man at the average Canadian height of 178 cm would weigh 58.5 kg for a BMI of 18.50 and 79.1 kg for a BMI of 25.0, representing an increase of 20.6 kg (45.4 pounds). In these two examples, to move up by one BMI point, the woman would have to gain 2.6 kg (5.7 pounds), while the man would have to gain 3.1 kg (6.8 pounds). Given the results in Figure 1, this suggests that men under age 45 and women under age 55 may be putting on approximately 0.45 kg or one pound per year, which levels off in the middle-aged groups and begins to reverse in the oldest age groups.

The regression models suggest that there are factors associated with change in BMI, but much of the variance remains unexplained, and the models must be interpreted with caution due to the wide confidence intervals associated with many of the estimates. Nevertheless, the findings tend to be consistent with previous research, although there are some notable exceptions. For example, several studies have noted an association between increased weight and a variety of comorbid conditions [3,11] as well as the number of comorbid conditions [11], which was not observed in the current study. This could in part be because comorbidities may have been less severe than is typical for many diseases, as very sick subjects tended not to participate or to drop out of the cohort. Moreover, previously noted associations between increased weight and lower income [10,22] were not found. Activity restriction also had little effect, despite previous research to the contrary [22], but that may be because other variables such as change in sedentary hours and change in participation in regular activity are also in our models. Level of pain also produced little in the way of interesting results, other than for women who noted moderate to severe pain that prevented some activities.

Our data did suggest a moderate effect of region, with the central region most likely to show an increase in BMI. One previous Canadian study noted almost no effect of region [22], but a study of region in the USA did note higher rates of obesity in the South Central and Northeast Central regions as compared to New England, the Atlantic regions, Mountain and Pacific regions [17]. Younger participants gained more weight than older participants, and the impact of menopause was small but in the expected direction [24], with more weight gain in those who were already menopausal at baseline.

Current smokers had less gain than those who had never smoked or had recently quit, particularly for women, which is consistent with other research [5,22], while the results for alcohol intake suggest that higher levels in intake are associated with small declines in weight, which differs from previous findings [22]. However, it should be noted that alcohol consumption in this sample was relatively low, with a median of 0.2 drinks per week for the women and 2.0 per week for the men.

All education levels below the University level were associated with more weight gain, which was more pronounced for women and consistent with past findings [5,10,18]. Number of children appeared to have little effect on weight change for women, which has also been noted by others [26], despite widely held beliefs to the contrary [25,26]. However, this may be because many respondents had their children long before the baseline measurement, and parity may therefore have contributed to baseline weight rather than change in weight.

The ability to assess the effect of behaviour change, and its impact on change in weight, is one of the strengths of this study. Compared to those who did not participate in physical activity but do now, all other groups showed small BMI gains, supporting other research that demonstrated a healthy effect of increased activity on BMI [10,29]. Moreover, compared to those who went from a high to a low number of sedentary hours, the other groups showed small gains in BMI. Those who indicated that their level of happiness had declined or stayed the same saw small decreases in BMI compared with those whose level of happiness had improved. Finally, a decrease in self-rated health, which has been associated with higher BMI in other research [30], was associated with increased BMI in women but not in men. For men, increase in BMI tended to be associated with a perception of improved general health.

One limitation to this study was the loss to follow-up, as only 90.7% of the baseline sample could be included even when using multiple imputation. Moreover, multiple imputation also has assumptions and limitations. For example, the technique assumes that the baseline data were sufficiently detailed to predict year 5 weights, and that, given the information available for predicting missing year 5 weights, those who participated are similar at baseline to those who did not participate [31]. It is possible that despite adjusting for baseline characteristics, the non-respondents at year 5 were different from those with complete data. However, inclusion of the imputed data was considered to be preferable to simply basing all estimates on those with complete data at both time points, as at least some bias adjustment is preferable to none.

Caution must be used when interpreting any results based on BMI data. While BMI is a commonly used indicator of weight category, it is a composite measure that is unable to distinguish between fat and lean tissue [20]. Moreover, the BMI cut-points for overweight and obese subjects may need to be adjusted for certain non-white people, as well as the elderly [2,10]. Finally, waist circumference, which is also an important indication for assessing obesity-related health risk [2,10,32], was not measured in the CaM*os *cohort.

## Conclusion

This study provides evidence that Canadians have a higher BMI than that which is currently recommended for optimal health. Moreover, weight appears to be increasing in younger age groups, although this reverses after age 65 in both men and women. These findings suggest that a substantial number of people may be putting their health at risk due to being overweight or obese. Although there is a tendency to stay in the same weight category, this increase in BMI over time underscores the need for public health efforts aimed at combating the current epidemic of obesity, especially in younger men and women.

## Competing interests

The author(s) declare that they have no competing interests.

## Authors' contributions

WMH, CB, SIB, JCP, MH and TT participated in the conception and design of this CaMos ancillary study; CL, CB and LJ were responsible for the analysis; all were involved in the interpretation of the findings and drafting the manuscript; all have read and approved the final manuscript.

## Appendix 1: CaM*os *Research Group

CaM*os *Coordinating Centre, McGill University, Montreal, Quebec: David Goltzman (principal investigator), Alan Tenenhouse (principal investigator emeritus), Suzette Poliquin (national coordinator), Suzanne Godmaire (research assistant), Claudie Berger (study statistician), Lawrence Joseph (consultant statistician).

Memorial University, St. John's Newfoundland: Carol Joyce (director), Christopher Kovacs (co-director), Emma Sheppard (coordinator).

Dalhousie University, Halifax, Nova Scotia: Susan Kirkland, Stephanie Kaiser (co-directors), Barbara Stanfield (coordinator).

Laval University, Quebec City, Quebec: Jacques P. Brown (director), Louis Bessette (co-director), Marc Gendreau (coordinator).

Queen's University, Kingston, Ontario: Tassos Anastassiades (director), Tanveer Towheed (co-director), Barbara Matthews (coordinator).

University of Toronto, Toronto, Ontario: Bob Josse (director), Tim Murray (co-director), Barbara Gardner-Bray (coordinator), Nancy Kreiger (principal investigator).

McMaster University, Hamilton, Ontario: Jonathan D. Adachi (director), Alexandra Papaioannou (co-director), Laura Pickard (coordinator).

University of Saskatchewan, Saskatoon, Saskatchewan: Wojciech P. Olszynski (director), K. Shawn Davison (co-director), Jola Thingvold (coordinator).

University of Calgary, Calgary, Alberta: David A. Hanley (director), Jane Allan (coordinator).

University British Columbia, Vancouver, British Columbia: Jerilynn C. Prior (director), Yvette Vigna (coordinator).

## Pre-publication history

The pre-publication history for this paper can be accessed here:



## References

[B1] Health Canada (2003). Canadian Guidelines for Body Weight Classification in Adults.

[B2] Gilmore J (1999). Body Mass Index and Health. Statistics Canada Catalogue 82-003. Health Reports.

[B3] Lau DCW (1999). Call for action: preventing and managing the expansive and expensive obesity epidemic. CMAJ.

[B4] Torrance GM, Hooper MD, Reeder BA (2002). Trends in overweight and obesity among adults in Canada (1970 – 1992): evidence from national surveys using measured height and weight. Int J Obesity.

[B5] Tremblay MS, Katzmarzyk PT, Willms JD (2002). Temporal trends in overweight and obesity in Canada, 1981 – 1996. Int J Obesity.

[B6] Katzmarzyk PT (2002). The Canadian obesity epidemic, 1985 – 1998. CMAJ.

[B7] Flegal KM, Carroll MD, Ogden CL, Johnson CL (2002). Prevalence and trends in obesity among US adults, 1999–2000. JAMA.

[B8] Hedley AA, Ogden CL, Johnson CL, Carroll MD, Curtin LR, Flegal KM (2004). Prevalence of overweight and obesity among US children, adolescents and adults, 1999–2002. JAMA.

[B9] Raine KD (2002). Overweight and Obesity in Canada: A Population Health Perspective.

[B10] Trakas K, Lawrence K, Shear NH (1999). Utilization of health care resources by obese Canadians. CMAJ.

[B11] Tjepkema M Adult obesity in Canada: Measured height and weight. Statistics Canada. http://www.statcan.ca/english/research/82-620-MIE/2005001/articles/adults/aobesity.htm.

[B12] World Health Organization (2000). Obesity : Preventing and Managing the Global Epidemic. Report of a WHO Consultation on Obesity.

[B13] Ogden CL, Carroll MD, Flegal KM (2003). Epidemiologic trends in overweight and obesity. Endocrinol Metab Clin North Am.

[B14] Katzmarzyk PT, Craig CL, Bouchard C (2001). Underweight, overweight and obesity: relationships with mortality in the 13-year follow-up of the Canadian Fitness Survey. J Clin Epi.

[B15] Katzmarzyk PT, Ardern CI (2004). Overweight and obesity mortality trends in Canada, 1985–2000. Can J Public Health.

[B16] Flegal KM, Graubard BI, Williamson DF, Gail MH (2005). Excess deaths associated with underweight, overweight and obesity. JAMA.

[B17] American Society of Plastic Surgeons (2006). The obesity epidemic. Plastic and Reconstructive Surgery.

[B18] Katzmarzyk PT, Janssen I (2004). The economic costs associated with physical activity and obesity in Canada: an update. Can J Appl Physiol.

[B19] De Laet C, Kanis JA, Odén A, Johanson H, Johnell O, Delmas P, Eisman JA, Kroger H, Fujiwara S, Garnero P, McCloskey EV, Mellstrom D, Melton LJ, Meuniere PJ, Pols HA, Reeve J, Silman A, Tenenhouse A (2005). Body mass index as a predictor of fracture risk: A meta-analysis. Osteoporosis International.

[B20] Heo M, Faith MS, Pietrobelli A (2002). Resistance to change of adult body mass index. Int J Obesity.

[B21] Katzmarzyk PT, Perusse L, Malina RM, Bouchard C (1999). Seven-year stability of indicators of obesity and adipose tissue distribution in the Canadian population. Am J Clin Nutr.

[B22] Le Petit C, Berthelot JM (2005). Obesity: A Growing Issue. In Healthy today, health tomorrow? Findings from the National Population Health Survey. Statistics Canada Catalogue 82–618-MWE2005003.

[B23] Kreiger N, Tenenhouse A, Joseph L, MacKenzie T, Poliquin S, Brown J, Prior JC, Rittmaster R (1999). Research Notes: The Canadian Multicentre Osteoporosis Study (CaM*os*): Background, Rationale, Methods. Can J Aging.

[B24] Lovejoy JC (2003). The menopause and obesity. Primary Care.

[B25] Wolfe WS, Sobal J, Olson CM, Frongillo EA, Williamson DF (1997). Parity-associated weight gain and its modification by sociodemographic factors: A prospective analysis in US women. Int J Obes Rel Metabolic Dis.

[B26] Lee SK, Sobal J, Frongillo EA, Olson CM, Wolfe WS (2005). Parity and body weight in the United States: differences by race and size of place of residence. Obes Res.

[B27] Rubin D (1987). Multiple Imputation for Non-response in Surveys.

[B28] Park AJ, Bloom SR (2005). Neuroendocrine control of food intake. Curr Opin Gastroenterol.

[B29] Pi-Sunyer FX The obesity epidemic: Pathophysiology and consequences of obesity. Obesity Research.

[B30] Kaplan MS, Huguet N, Newsom JT, McFarland BH, Lindsay J (2003). Prevalence and correlates of overweight and obesity among older adults: findings from the Canadian National Population Health Survey. J Gerontol A Biol Sci Med Sci.

[B31] Kmetic A, Joseph L, Berger C, Tenenhouse A (2002). Multiple imputation to account for missing data in a survey: estimating the prevalence of osteoporosis. Epidemiology.

[B32] Douketis JD, Paradis G, Keller H, Martineau C (2005). Canadian guidelines for body weight classification in adults: application in clinical practice to screen for overweight and obesity and to assess disease risk. CMAJ.

